# Draft genome sequence of strain HIMB100, a cultured representative of the SAR116 clade of marine *Alphaproteobacteria*

**DOI:** 10.4056/sigs.1854551

**Published:** 2011-12-23

**Authors:** Jana Grote, Cansu Bayindirli, Kristin Bergauer, Paula Carpintero de Moraes, Huan Chen, Lindsay D’Ambrosio, Bethanie Edwards, Beatriz Fernández-Gómez, Mariam Hamisi, Ramiro Logares, Dan Nguyen, Yoshimi M. Rii, Emily Saeck, Charles Schutte, Brittany Widner, Matthew J. Church, Grieg F. Steward, David M. Karl, Edward F. DeLong, John M. Eppley, Stephan C. Schuster, Nikos C. Kyrpides, Michael S. Rappé

**Affiliations:** 1 Center for Microbial Oceanography: Research and Education, University of Hawaii, Honolulu, Hawaii, USA; 2 Hawaii Institute of Marine Biology, SOEST, University of Hawaii, Kaneohe, Hawaii, USA; 3 Plymouth Marine Laboratory, University of East Anglia, Norwich, UK; 4 Department of Marine Biology, University of Vienna, Vienna, Austria; 5 Instituto Oceanografico, Universidade de Sao Paulo, Sao Paulo, Brazil; 6 Environmental Sciences Institute, Florida A&M University, Tallahassee, Florida, USA; 7 Department of Marine Sciences, University of North Carolina, Chapel Hill, North Carolina, USA; 8 Scripps Institution of Oceanography, University of California, San Diego, La Jolla, California, USA; 9 Departament de Biologia Marina i Oceanografia, Institut de Cièncias del Mar, CMIMA, CSIC, Barcelona, Spain; 10 School of Natural Sciences and Mathematics, The University of Dodoma, Dodoma, Tanzania; 11 Département de Sciences Biologiques, Universitéde Montréal, Montréal, Canada; 12 Department of Oceanography, SOEST, University of Hawaii, Honolulu, Hawaii, USA; 13 Australian Rivers Institute, Griffith University, Queensland, Australia; 14 Department of Marine Sciences, University of Georgia, Athens, Georgia, USA; 15 Department of Ocean, Earth and Atmospheric Sciences, Old Dominion University, Norfolk, Virginia, USA; 16 Department of Civil and Environmental Engineering, Massachusetts Institute of Technology, Cambridge, Massachusetts, USA; 17 Center for Comparative Genomics and Bioinformatics, Pennsylvania State University, University Park, Pennsylvania, USA; 18 Department of Energy Joint Genome Institute, Walnut Creek, California, USA

**Keywords:** marine bacterioplankton, genome, proteorhodopsin, SAR116, *Rhodospirillaceae*

## Abstract

Strain HIMB100 is a planktonic marine bacterium in the class *Alphaproteobacteria*. This strain is of interest because it is one of the first known isolates from a globally ubiquitous clade of marine bacteria known as SAR116 within the family *Rhodospirillaceae*. Here we describe preliminary features of the organism, together with the draft genome sequence and annotation. This is the second genome sequence of a member of the SAR116 clade. The 2,458,945 bp genome contains 2,334 protein-coding and 42 RNA genes.

## Introduction

HIMB100 is a taxonomically uncharacterized marine bacterial strain isolated from surface seawater collected off the coast of Oahu, Hawaii in the subtropical Pacific Ocean [1]. It is of significant interest because it belongs to a 16S rRNA gene clade of marine *Alphaproteobacteria* known as SAR116, which was first described by Mullins *et al.* in 1995 [2] and has since been found to be widespread in the global surface ocean based on cultivation-independent surveys of marine bacterioplankton [3-9]. The first cultured strain of this clade was isolated from surface waters of the Pacific Ocean off the coast of Oregon, USA, in 2007 [10]. In 2010, the genome sequence of *Candidatus* Puniceispirillum marinum IMCC1322, a cultivated member of the SAR116 clade isolated from the East Sea in the Western Paciﬁc Ocean (Sea of Japan), was reported [11]. Here we present a preliminary set of features for strain HIMB100 (Table 1), together with a description of the complete genomic sequencing and annotation, as well as a preliminary comparative analysis with the complete genome of *Candidatus* P. marinum IMCC1322.

**Table 1:** Classification and general features of strain HIMB100 according to the MIGS recommendations [12]

**MIGS ID**	**Property**	**Term**	**Evidence code**
	Current classification	Domain *Bacteria*	TAS [13]
		Phylum *Proteobacteria*	TAS [14]
		Class *Alphaproteobacteria*	TAS [15,16]
		Order *Rhodospirillales*	TAS [17,18]
		Family *Rhodospirillaceae*	TAS [17,18]
		Genus not assigned	
		Species not assigned	
		Type strain HIMB100	IDA
	Gram stain	negative	NAS
	Cell shape	spiral-shaped	IDA
	Motility	unknown	
	Sporulation	non-sporulating	NAS
	Temperature range	mesophilic	IDA
	Optimum temperature	unknown	
	Carbon source	ambient seawater DOC	TAS [1]
	Energy source	chemoorganoheterotrophic	NAS
MIGS-6	Habitat	sea water	
MIGS-6.3	Salinity	~35.0 ‰	NAS
MIGS-22	Oxygen	aerobic	NAS
MIGS-15	Biotic relationship	free-living	TAS [1]
MIGS-14	Pathogenicity	none	NAS
MIGS-4	Geographic location	Kaneohe Bay, Hawaii, subtropical Pacific Ocean	TAS [1]
MIGS-5	Sample collection time	18 May 2005	TAS [1]
MIGS-4.1	Latitude	21.44	TAS [1]
MIGS-4.2	Longitude	-157.78	TAS [1]
MIGS-4.3	Depth	~1 m	TAS [1]

Evidence codes - IDA: Inferred from Direct Assay (first time in publication); TAS: Traceable Author Statement (i.e., a direct report exists in the literature); NAS: Non-traceable Author Statement (i.e., not directly observed for the living, isolated sample, but based on a generally accepted property for the species, or anecdotal evidence). These evidence codes are from the Gene Ontology project [19]. If the evidence code is IDA, the property was directly observed by one of the authors or an expert mentioned in the acknowledgements.

## Classification and features

Strain HIMB100 was isolated by a high-throughput, dilution-to-extinction approach [20] from seawater collected off the coast of Hawaii, USA, in the subtropical North Pacific Ocean, and bore an identical 16S rRNA gene sequence to three other isolates obtained from the same study [1]. All four strains were isolated in seawater sterilized by tangential flow filtration and amended with low concentrations of inorganic nitrogen and phosphorus (1.0 µM NH_4_Cl, 1.0 µM NaNO_3_, and 0.1 µM KH_2_PO_4_). Repeated attempts to cultivate the isolates on solidified culture media or in artificial seawater media failed. In addition, preliminary attempts have failed to identify amendments to the seawater-based culture medium that would increase the abundance of cells in culture above ca. 1 ×10^6^ cells ml^-1^.

**Figure 1:**
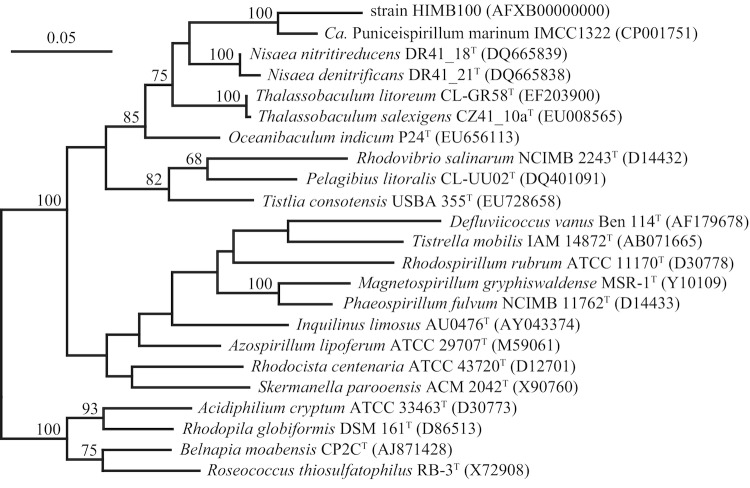
Phylogenetic tree based comparisons between 16S rRNA gene sequences from strain HIMB100, *Candidatus* Puniceispirillum marinum IMCC1322, and type strains of related species within the family *Rhodospirillaceae*. Sequence selection and alignment improvements were carried out using the ‘All-Species Living Tree’ project database [21] and the ARB software package [22]. The tree was inferred from 1,206 alignment positions using the RAxML maximum likelihood method [23]. Support values from 100 bootstrap replicates, determined by RAxML [24], are displayed above branches if larger than 60%. The scale bar indicates substitutions per site.

Phylogenetic analyses based on 16S rRNA gene sequence comparisons revealed strain HIMB100 to be closely related to a large number of environmental gene clones obtained almost exclusively from seawater. For example, alignment of HIMB100 against the Silva release 104 reference database (512,037 high quality bacterial 16S rRNA sequences >1200 base pairs in length, released October 2010) revealed 554 entries that belong to the same phylogenetic lineage within the *Alphaproteobacteria*. Of these, only one originated from a cultivated isolate (*Candidatus* P. marinum IMCC1322), and all 554 entries derived from either seawater or the marine environment. The 16S rRNA gene sequence from Oregon coast strain HTCC8037 was 98.0% similar to that of strain HIMB100, but it does not appear in the Silva reference database because it is a partial sequence of 884 nucleotides in length [10]. In phylogenetic analyses with taxonomically described members of the *Alphaproteobacteria*, strain HIMB100 and *Candidatus* P. marinum IMCC1322 (94.1% similar) formed a monophyletic lineage within the family *Rhodospirillaceae* (Figure 1). The 16S rRNA gene of strain HIMB100 was most similar to the type strains of *Nisaea denitrificans* (90.3%), *N. nitritireducens* (89.9%), and *Thalassobaculum salexigens* (89.3%), which were all isolated from surface seawater of the northwestern Mediterranean Sea [25,26], *T. litoreum* (89.5%), isolated from coastal seawater off of Korea [27], and *Oceanibaculum indicum* (89.4%), isolated from a polycyclic aromatic hydrocarbon-degrading consortium that was enriched from a deep-seawater sample collected from the Indian Ocean [28].

**Figure 2:**
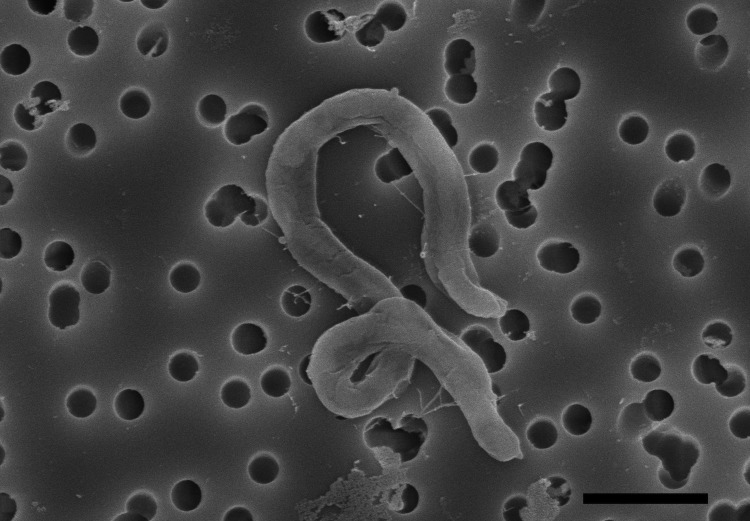
Scanning electron micrograph of strain HIMB100. Scale bar corresponds to 1 μm.

Cells of strain HIMB100 are long, thin spiral-shaped rods (0.3 x 1-5 μm) when in exponential growth (Figure 2). Because it is able to grow in media consisting solely of sterile seawater with added inorganic nitrogen and phosphorus in the light or dark, HIMB100 is presumed to grow chemoheterotrophically by oxidizing compounds in the dissolved organic carbon pool of natural seawater. A summary of other known preliminary features is shown in Table 1.

### Chemotaxonomy

No cellular fatty acids profiles are currently available for strain HIMB100, nor have any been reported for other cultivated members of the SAR116 clade.

## Genome sequencing and annotation

### Genome project history

Strain HIMB100 was selected for sequencing because of its phylogenetic affiliation with a widespread lineage of marine bacteria that is significantly underrepresented in culture collections. The genome project is deposited in the Genomes OnLine Database (GOLD) as project Gi06671, and the complete genome sequence in GenBank as accession number AFXB00000000 [Table 2]. A summary of the main project information is shown in Table 2.

**Table 2:** Genome sequencing project information

**MIGS ID**	**Property**	**Term**
MIGS-31	Finishing quality	Finished
MIGS-28	Libraries used	one standard 454 pyrosequence titanium library
MIGS-29	Sequencing platforms	454 GS FLX Titanium
MIGS-31.2	Fold coverage	132 × pyrosequence
MIGS-30	Assemblers	Newbler version 2.3
MIGS-32	Gene calling method	Prodigal 1.4, GenePRIMP
	Genbank ID	AFXB00000000
	Genbank Date of Release	November 10, 2011
	GOLD ID	Gi0667
	Database: IMG	2503113005
	NCBI taxon ID	281031
MIGS-13	Source material identifier	HIMB100
	Project relevance	environmental

### Growth conditions and DNA isolation

Strain HIMB100 was grown at 27° C in 50 L of coastal Hawaii seawater sterilized by tangential flow filtration [1] and supplemented with (final concentration) 10 µM NH_4_Cl, 1.0 µM KH_2_PO_4_, 1.0 µM L-serine, 1.0 µM L-methionine, 10 mM FeCl_3_, 0.1 µM betaine, 0.001% (wt/vol) of D-ribose, D-glucose, succinic acid, pyruvic acid, glycerol, and N-acetyl-D-glucosamine, 0.002% (vol/vol) ethanol, and Va vitamin mix at a 10^-3^ dilution [20]. Cells from the liquid culture were collected on a membrane filter, and DNA was isolated from the microbial biomass using a standard phenol/chloroform/isoamyl alcohol extraction protocol. A total of ca. 12 μg of DNA was obtained.

### Genome sequencing and assembly

The genome of strain HIMB100 was sequenced at the Pennsylvania State University Center for Comparative Genomics and Bioinformatics (University Park, PA, USA) using the 454 GS FLX Ti platform of 454 Life Sciences (Branford, CT, USA). The sequencing library was prepared according to the 454 instructions from genomic DNA of strain HIMB100. Sequencing was carried out on a full 454 picotiter plate, yielding 1,342,353 reads with an average length of 415 bp, totaling 556 Mbp. Pyrosequencing reads were assembled using the Newbler assembler version 2.3, resulting in 10 contigs of 2,458,945 bp. Sequencing provided 132 × coverage of the genome.

### Genome annotation

Genes were identified using Prodigal [29] as part of the genome annotation pipeline in the Integrated Microbial Genomes Expert Review (IMG-ER) system [30]. The predicted coding sequences were translated and used to search the National Center for Biotechnology Information (NCBI) nonredundant database, UniProt, TIGRFam, Pfam, PRIAM, KEGG, COG, and InterPro databases. The tRNAScanSE tool [31] was used to find tRNA genes, whereas ribosomal RNAs were found by using the tool RNAmmer [32]. Other non-coding RNAs were identified by searching the genome for the Rfam profiles using INFERNAL (v0.81) [33]. Additional gene prediction analysis and manual functional annotation was performed within IMG-ER.

## Genome properties

**Figure 3:**
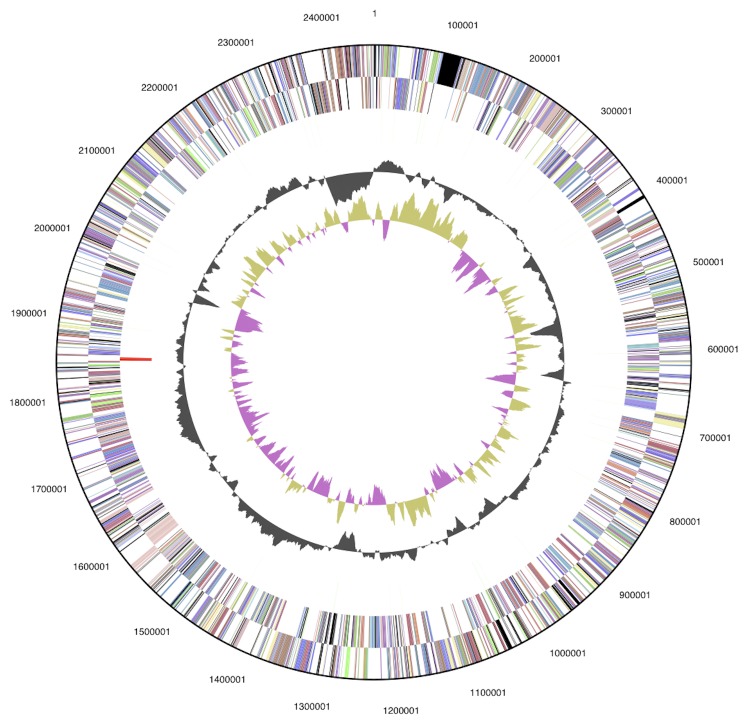
Graphic circular map of the HIMB100 genome. From outside to the center: Genes on forward strand (colored by COG categories), Genes on reverse strand (colored by COG categories), RNA genes (tRNAs green, rRNAs red, other RNAs black), GC content, GC skew. The 10 contigs of the draft genome are ordered randomly.

The genome is 2,458,945 bp long and comprises 10 contigs ranging in size from 30,717 to 1,167490 bp, with an overall GC content of 50.48 % (Table 3 and Figure 3). Of the 2,376 genes predicted, 2,334 were protein coding genes, and 42 were RNAs. Most protein coding genes (82.0%) were assigned putative functions, while the remaining genes were annotated as hypothetical proteins. The distribution of genes into COG functional categories is presented in Table 4.

**Table 3:** Genome statistics

**Attribute**	**Value**	**% of total^a^**
Genome size (bp)	2,458,945	100.00
DNA coding region (bp)	2,260,613	91.91
DNA G+C content (bp)	1,241,171	50.48
Total genes	2,376	100.00
RNA genes	42	1.77
Protein-coding genes	2,334	98.23
Genes with function prediction	1,948	81.99
Genes assigned to COGs	1,873	78.83
Genes assigned to Pfam domains	1,957	82.37
Genes with signal peptides	733	30.43
Genes with transmembrane helices	504	21.21

a) The total is based on either the size of the genome in base pairs or the total number of protein coding genes in the annotated genome.

**Table 4:** Number of genes associated with the 25 general COG functional categories

**Code**	**Value**	**%age**^a^	**Description**
J	142	6.8	Translation
A	0	0	RNA processing and modification
K	77	3.7	Transcription
L	93	4.5	Replication, recombination and repair
B	2	0.1	Chromatin structure and dynamics
D	20	1.0	Cell cycle control, mitosis and meiosis
Y	0	0	Nuclear structure
V	16	0.8	Defense mechanisms
T	41	2.0	Signal transduction mechanisms
M	142	6.8	Cell wall/membrane biogenesis
N	41	2.0	Cell motility
Z	0	0	Cytoskeleton
W	0	0	Extracellular structures
U	40	1.9	Intracellular trafficking and secretion
O	89	4.3	Posttranslational modification, protein turnover, chaperones
C	149	7.1	Energy production and conversion
G	126	6.0	Carbohydrate transport and metabolism
E	250	12.0	Amino acid transport and metabolism
F	58	2.8	Nucleotide transport and metabolism
H	117	5.6	Coenzyme transport and metabolism
I	113	5.4	Lipid transport and metabolism
P	70	3.4	Inorganic ion transport and metabolism
Q	78	3.7	Secondary metabolites biosynthesis, transport and catabolism
R	271	13.0	General function prediction only
S	155	7.4	Function unknown
-	503	21.2	Not in COGs

a) The total is based on the total number of protein coding genes in the annotated genome.

## Genome comparisons with ***Candidatus* Puniceispirillum marinum **IMCC1322

The genome of one other member of the SAR116 clade, *Candidatus* P. marinum IMCC1322, was recently sequenced [11]. This genome is 2,753,527 bp in length (295 Kbp longer than HIMB100), arranged in a single chromosome, and possesses a G + C content similar to that of HIMB100 (48.85% vs. 50.48%). Although the genome of *Candidatus* P. marinum IMCC1322 is annotated with over 200 more genes than HIMB100 (2,582 genes vs. 2,376), it only encodes for 51 additional protein-coding genes with predicted function.

The predicted metabolic potentials encoded by the two genomes have many features in common. The genomes of both strains possess a lesion in the Embden-Meyerhoff-Parnas pathway in that they lack the enzyme 6-phosphofructokinase. However, the genomes of both strains possess two key enzymes of the Entner-Doudoroff pathway, phosphogluconate dehydratase and 2-keto-3-deoxy-phosphogluconate aldolase. The oxidative portion of the pentose phosphate pathway is incomplete in both strains; the genome of HIMB100 lacks a recognizable 6-phosphogluconolactonase, while the genomes of both strains lack a recognizable 6-phosphogluconate dehydrogenase. In addition, several genes of predicted biogeochemical importance are present in both strains, including proteorhodopsin and carotenoid biosynthesis genes, carbon monoxide dehydrogenase, dimethylsulfoniopropionate (DMSP) demethylase, and dimethylsulfoxide (DMSO) reductase. Genes for assimilatory sulfate reduction are incomplete in both genomes, and so it is hypothesized that exogenous reduced sulfur compounds, such as DMSP and DMSO, are likely to fill the requirement of sulfur for cellular growth. The genomes of both strains possess a high affinity inorganic phosphate transport system (pstSCAB), and encode a phosphate regulon sensor (phoU), phosphate starvation-inducible protein (phoH), and the phosphate regulon consisting of the phoB-phoR two-component system. Both genomes encode for ABC transporters for iron, glycine betaine/proline, zinc, sorbitol/mannitol, amino acids (branched-chain and general L-amino acids), sulfonate/nitrate/taurine and a heme exporter. Thiamine and alpha-glucoside transport systems were only identified within the genome of strain HIMB100, while ribose and putrescine transport systems were only identified within the genome of *Candidatus* P. marinum IMCC1322. Finally, two operons of potential ecological relevance show different distributions within the two genomes: the genome of strain HIMB100 possesses a seven-gene operon encoding all of the subunits and accessory proteins for urease that is completely lacking in the genome of *Candidatus* P. marinum IMCC1322, while the genome of *Candidatus* P. marinum IMCC1322 possesses 21 genes for cobalamin biosynthesis that are absent from the genome of strain HIMB100.
